# Did *Bartonella henselae* contribute to the deaths of two veterinarians?

**DOI:** 10.1186/s13071-015-0920-4

**Published:** 2015-06-12

**Authors:** Edward B. Breitschwerdt

**Affiliations:** Intracellular Pathogens Research Laboratory and the Center for Comparative Medicine and Translational Research, College of Veterinary Medicine, North Carolina State University, Raleigh, NC USA; College of Veterinary Medicine, North Carolina State University, 1060 William Moore Dr., Raleigh, NC 27607 USA

**Keywords:** Infection, Disease, Mortality, Cancer, Heart valve, Blood

## Abstract

*Bartonella henselae*, a flea-transmitted bacterium, causes chronic, zoonotic, blood stream infections in immunocompetent and immunocompromised patients throughout the world. As an intra-erythrocytic and endotheliotropic bacterium, *B. henselae* causes a spectrum of symptomatology ranging from asymptomatic bacteremia to fever, endocarditis and death. Veterinary workers are at occupational risk for acquiring bartonellosis. As an emerging, and incompletely understood, stealth bacterial pathogen, *B. henselae* may or may not have been responsible for the deaths of two veterinarians; however, recent evidence indicates that this genus is of much greater medical importance than is currently appreciated by the majority of the biomedical community.

The question “Did *Bartonella henselae* contribute to the deaths of two veterinarians?” will never be definitively answered; however, after months of asking myself this question, I decided to comment on the possibility that a recently discovered stealth bacteria [1] may have played a role in their deaths. If one were to organize a physician/scientist review of each patient’s medical record, examining the voluminous doctors’, nurses’, consultants’ notes and hundreds of laboratory results; if this team were to visit with wives, family, and friends left behind, and had access to the test results generated by our research group (see acknowledgements) prior to and after their respective deaths, I am of the opinion that this question could never be accurately or adequately answered.

Perhaps questions for which there are not definitive answers should never be asked, but if true, there would be no need for biomedical research. Unfortunately, many questions remain unanswered, a fact that is very hard for patients, family members, doctors, and diagnosticians to accept. Medicine remains “a practice,” based upon the best available science, the art of patient care, and a clinician’s attempts to manage symptoms and disease processes for which causation is elusive. Because of circumstances beyond everyone’s control, involvement of my research laboratory team was less than optimal due to the timing of specimen collection and type of samples available for *Bartonella* testing.

Voltaire, (1694 to 1778), stated: “Doctors put drugs of which they know little, into our bodies of which they know less, to treat diseases of which they know nothing at all.” Clearly, medical science and 21^st^ century patient care have advanced tremendously since Voltaire’s time; however, there are still major gaps in scientific knowledge that negatively impact patient outcomes. Specifically, our collective lack of “knowledge” negatively impacts directed therapy and effective management of the patient’s illness and deficiencies in medical knowledge related to the genus *Bartonella* continue to compromise patient care throughout the world.

It is my hope that this commentary will benefit future patients, particularly veterinary workers, who have been or will become infected with the emerging and incompletely understood, stealth bacterial pathogen; *Bartonella henselae*. The term “emerging infectious disease” has been abused and to an extent overused in recent years, often in association with efforts to influence the appropriation of research funding. During the past two decades, bartonellosis has clearly earned the designation “an emerging infectious disease.” The genus *Bartonella* has expanded from two known species prior to 1992 to at least 34 *Bartonella* species in 2015. Medically, fifteen *Bartonella* species have been associated with a spectrum of human illnesses; therefore, incompletely understood is another way to accurately describe this genus. The fascinating history of *Bartonella* has been summarized in recent reviews [2–4]; whereas the contemporary history of this genus is being written and rewritten as new knowledge is generated around the world. The past and more recent history of bartonellosis [1–4] is beyond the scope of this commentary. However, a brief historical perspective is necessary to put the lives and deaths of these veterinarians into context. Prior to the recognition that bacillary angiomatosis and peliosis hepatis were caused by *Bartonella quintana* or *B. henselae* in AIDS patients [5, 6], bartonellosis was not a differential diagnosis for sick humans or other animals throughout most of the world [1, 3]. Prior to the AIDS epidemic, indigenous infection with a *Bartonella* sp. had never been diagnosed by a physician or veterinarian in North America. Not surprisingly, clinicians cannot diagnose an as yet “unknown or undiscovered” infectious agent. Importantly, the history of *Bartonella* spp. infections substantially pre-dates AIDS. *Bartonella bacilliformis*, transmitted by sandflies, caused Oroya Fever and verruga peruana in indigenous Peruvian Indians hundreds of years ago; infected Spanish conquistadors, and caused highly fatal illness in immigrant workers building the Trans Peruvian railroad [2–4]. Purportedly, one worker died of hemolytic anemia (Oroya Fever) for each railroad tie that was laid. A second *Bartonella* species, *B. quintana*, transmitted by the human body louse, was a major cause of morbidity and mortality during the World Wars [2–4], and continues to cause human suffering and death among a spectrum of people (urban trench fever), particularly individuals experiencing human body louse exposure in association with wars and famine, or those living in poverty and abusing drugs in modern day cities. Since the “rediscovery” of *Bartonella* species infections in AIDS patients, numerous new species have been implicated as a cause of illnesses in immunocompromised and immunocompetent patients [2, 7, 8]. Despite a dramatic expansion of medical and microbiological knowledge related to the genus *Bartonella* [1, 3, 6], there are still major knowledge gaps involving clinically relevant questions.

## Findings

Due to their lengthy illnesses, frequent medical evaluations, and involvement of numerous medical specialists and medical centers throughout the United States, only a brief, superficial overview of each veterinarian’s illness is possible. As an example of the medical complexity, veterinarian #1 was transferred among six different hospitals during the last 5 months of his life (October 2012 through February 2012). Selected historical and demographic information is summarized (Table 1). When I entered the University of Georgia, College of Veterinary Medicine in 1970, Veterinarian #1 was a professor. I will always remember him as a caring, enthusiastic, outgoing and intelligent teacher. Before I graduated, he left the University and started a companion animal veterinary practice, where he worked for the remainder of his career. On April 11th, 2011, I received this email communication from him: “Ed. 15 years ago, my cardiologist told me I was in an "elite group" of individuals as pertaining to cardiovascular fitness. Last week, we discovered I need a mitral, aortic and tricuspid valve replacement to be done this Friday. Remembering some of your articles, I believe you stated that the number 1 cause of culture-negative endocarditis in humans was bartonellosis. I have been hospitalized twice in the last 2 years for severe pneumonia. Do you think that is something I should check on and if so, what samples could I send to you to attempt culture or PCR. I hate to impose on you like this, but my cardiologist does not give it much weight nor does my family practitioner. Hope all is well with you and family.”

**Table 1:** Historical and demographic findings extracted from the study questionnaire provided by the two veterinarians infected with *Bartonella henselae*

Parameter	Veterinarian #1	Veterinarian #2
Age	67	63
Sex	Male	Male
Ethnicity	Caucasian	Caucasian
Residence	Suburban	Suburban
Number of children	Daughter	2 Sons
Employment Status	Full-time employed	Full-time employed
Occupation	Veterinarian	Veterinarian
Length of time at current position	44 years	37 years
Medical coverage	Yes	Yes
Have you been diagnosed with infectious disease	Yes- pneumonia	Yes - childhood diseases, influenza
Have you been diagnosed with a chronic illness	Yes- approximately 2 years duration	Yes – mild colitis, gastric reflux
Allergies	Yes – Environmental	Yes – mild seasonal rhinitis
Has experienced persistent illness	Yes	No
Self-described health	Infrequently ill	Healthy
**History of symptoms**:		
Fatigue	Yes	No
Difficulty remembering	Yes	No
Disoriented (confused by time or place)	Yes	No
Eye Pain	Yes	No
Difficulty sleeping (insomnia)	Yes	No
Chronic fatigue	Yes	No
Bladder dysfunction	Yes	No
Fever	Yes	No
Mental confusion (disordered thoughts)	Yes	No
Blurred vision	Yes	No
Balance problems	Yes	No
Shortness of breath	Yes	No
Muscle weakness	Loss of sensation or numbness in legs	Legs – weak left lower leg since ICE chemotherapy – symptoms almost gone
Ability to perform the same activities of daily living prior to illness?	No	Yes, until medical leave began July 2012
Ability to perform same employment/school activities prior to illness?	No	Yes, until medical leave began July 2012
Self-reported signs of symptoms	Waxing/waning	Improving – with current cancer treatment
**History of Physician evaluations**:		
General Practitioner	Yes	Yes
Pulmonologist	Yes	No
Orthopedist	Yes	Yes
Urologist	Yes	No
Internal Medicine	Yes	Yes
Infectious Disease	Yes	Yes
Gastroenterologist	Yes	Yes
Chiropractor	Yes	Yes
Ophthalmologist	Yes	Yes
Dermatologist	Yes	Yes
Dentist	Yes	Yes
Cardiologist	Yes	Yes
Neurologist	Yes	No
Psychiatrist/Psychologist	Yes	No
Physical Therapist	Yes	No
Podiatrist	Yes	No
Allergist	Yes	Yes
Acupuncturist	No	Yes
Ear/Nose/Throat	No	Yes
Oncologist	No	Yes
**Family Medical History**		
Heart Disease	Self + 2 Brothers	Father
Cancer	Sister – Mammary, Brother – Squamous Cell Carcinoma	Mother – Colon Cancer
Hypertension	Self + 2 Brothers	Father + Mother
Asthma	Self + Brother	
Stroke	Father	Mother
Substance Abuse	Brother	
Allergies	Self + Mother	Self
Diabetes		Father
**Animal contact**:		
Dog	Yes – Indoor/Outdoor	Yes – Indoor/Outdoor
Cat	Yes – Indoor/Outdoor	Yes – Indoor/Outdoor
Bird	Yes – Indoor	Yes – Indoor
Horse	Yes	Yes – Outdoor
Rodent	Yes	Yes, Hampster – Indoor
Reptile	No	Yes – Indoor
Fish	No	Yes
Ferret	No	Yes
Age of Exposure	0-60+	0-60+
Allow pets to sleep in same bed as owner	Yes	Yes – cats only
Allow pets to lick hands, face, feet, etc.	Yes	Yes
Frequent exposure (>1 time per month) to other animals	Yes – Dog, Cat	Yes – Dog, Cats for 29 years.
Length of exposure to other animals	>10 years	No
Exposure to production animals	No	First 8 years post-graduation, treated farm animals
**Animal bites or scratches**		
Dog	Yes – since 1960	No
Cat	Yes – since 1970	Yes – since 1975
Bird	No	Yes – 1980
Horse	No	Yes – 1975
Reptile	No	Yes – 1980
Rabbit	No	Yes – 1975
Rodent	No	Yes – 1980
**Outdoor exposure at least once a year**	Wildlife rescue/rehabilitation, Gardening	Hiking, Hunting, Gardening, Cycling, Outdoor water sports
**Antibiotics last 12 months**:		
Ciprofloxacin	Current	No
Doxycycline	Current	Current *Rigors & fevers stopped with start of Doxycycline
cefixime	June-2011	No
Fluoroquinolones	Yes	Yes
Cephalosporins	No	Yes
**Corticosteroids in past 12 months**:	Yes	Yes
Medrol Pak	12/11/2011-12/26/2011	No
Flonase	6/2011-current	No
Oral Prednisone	No	Yes
IV Prednisone	No	Yes
IV Dexamethasone	No	Yes
**Other medications in past 12 months**:		
Quinapril	2000-current	No
Qmlodipine	2000-current	No
Escitalopram	1980-current	No
Primidone	2007-current	No
Coumadin	2011-current	No
Aspirin 81 mg	1990-current	No
Modafinil	2010-current	No
Enalipril	No	Yes
Rosuvastatin calcium	No	Yes
Oral Antiviral	No	Yes
Oral Antifungal	No	Yes
**Insect Exposure**:		
Fleas	Yes	Yes
Ticks	Yes	Yes
Biting Flies	Yes	Yes
Mosquitoes	Yes	Yes
Spiders	Yes	Yes
Scabies	Yes	Yes
**Travel outside of state of residence**		
Northwest US	Yes	Yes
Northeast US	Yes	Yes
Southwest US	Yes	Yes
Southeast US	Yes	Yes
**Animal Contact during Travel Within US**		
Northwest US	Yes	No
Northeast US	Yes	No
Southwest US	Yes	Yes
Southeast US	Yes	No
**Non continental US & International Travel**		
Alaska	Yes	Yes
Mexico	Yes	Yes
Central America	Yes	Yes
Carribean	Yes	Yes
Canada	Yes	Yes
Australia/New Zealand/Fiji	No	Yes
Europe	No	Yes
**Animal Contact during Travel Outside Non continental US**		
Alaska	Yes	No
Mexico	No	No
Central America	Yes	No
Carribean	Yes	No
Canada	No	No
Pet Travel	No	No
**Specialist Doctor Visits**:		
Chronic mild colitis	No	Yes
T Cell Lymphoma	No	Yes
Hyperlipidemia	No	Duration 15 years – under control with statin

Although our paths had crossed occasionally during the ensuing 38 years, this email request initiated his entry into our Institutional Review Board approved research study (North Carolina State University, 164-08-05). This man was in good health until June 2010, when he lost consciousness, fell and was diagnosed with pneumonia. In April 2011, he had mitral valve replacement surgery, closure of a patent foramen ovale, 2-vessel bypass, bilateral maze procedure, radio frequency ablation, ligation of the left atrial appendage and pacemaker placement. Based upon self-reporting in a standardized questionnaire completed by all participants in our IRB-approved study, progressive symptoms including fatigue, disorientation, blurred vision, balance problems, difficulty remembering, eye pain, insomnia, muscle weakness, loss of sensation or numbness in the legs, and shortness of breath developed postoperatively in 2011. These non-specific symptoms are often reported by veterinary workers infected with one or co-infected with more than one *Bartonella* sp. [9–14]. Veterinarians, veterinary technicians, animal handlers, and groomers appear to be occupationally at risk groups for *Bartonella* spp. infections [9, 10]. Because of their frequent exposure to arthropod-infested, *Bartonella* spp. bacteremic animals, we have suggested that veterinary workers represent a sentinel, research study population for clarifying the medical importance of the genus *Bartonella* [15, 16]. From the context of research priorities and research funding, this suggestion has been ignored by policy makers and governmental agencies in the United States.

Relapsing fever of unknown origin (FUO) with temperatures as high as 104 °F, developed in October 2011 (6 months after surgery). Despite various antibiotic therapies, febrile episodes, accompanied by periodic dizziness and increasingly severe muscle weakness, continued until his death on February 24, 2013. *Bartonella* spp. are a cause of FUO and culture-negative endocarditis [17–20]. As reviewed by Chomel, *et al*. [17], the first description of human *Bartonella* endocarditis was published in 1993. Subsequently, *Bartonella* endocarditis was reported in cats, cows, dogs and sea otters [17].

Microbiological improvements in *Bartonella* sp. isolation methods and PCR amplification of organism-specific DNA sequences from surgically removed or autopsy obtained heart valves have resulted in the identification of *Bartonella* endocarditis cases throughout the world [17, 18]. Between January 23^rd^ and January 30^th^, 2012, multiple blood cultures were obtained after consultation with an infectious diseases physician, of which a subset was sent to our laboratory for *Bartonella* testing. FUO had persisted despite prior treatment with cefixime and concurrent administration of doxycycline and ciprofloxacin. When *Mycobacterium interjectum* grew in one blood culture processed by a commercial laboratory, the patient was treated with doxycycline and clarithromycin beginning in March 2012, followed by clofazimine and clarithromycin. *Mycobacterium interjectum*, a slow growing mycobacteria most often associated with immunosuppression, generally responds to antibiotic treatment. *Bartonella henselae* (SA2 genotype) was amplified and sequenced from one of three blood specimens submitted to our laboratory (Table 2). In September 2012, the patient’s fever pattern worsened with temperatures spiking to 105 °F, 2-3 times/week. Due to the poor response to antibiotics, an extensive workup was performed. Bone marrow and liver biopsies contained granulomatous inflammation, which occurs in association with *Bartonella*, *Mycobacterium* and other intracellular pathogens. Bronchoscopic lavage culture grew *M. interjectum* and *Eikenella corrodens*, after which treatment consisted of tigecycline and prednisolone (60 mg daily), which he had been taking for most of the previous year for potential sarcoidosis. In January 2013, the patient was admitted to National Jewish Health, for evaluation of severe muscular weakness, dyspnea on exertion, memory loss and fever of unknown origin. During the diagnostic evaluations the patient’s condition deteriorated resulting in transfer to the University of Colorado in Denver. After flying home to Florida, the patient was hospitalized at Bayfront Medical Center, after which he was transferred to Shands Hospital, University of Florida. On February 13^th^, 2013, after 13 hours of surgery for vegetative valvular endocarditis, valve replacement surgery, splenectomy, and pacemaker removal the patient never regained consciousness. The death certificate listed multi-organ failure, sepsis, and mycobacterial endocarditis as the cause of death. Nearly two years after his death, *B. henselae* with sequence identity of 527/527 bp with *B. henselae* Fizz, (Gen Bank accession number AF369526) was amplified and sequenced from a paraffin-embedded bone marrow specimen obtained in January 2012. The same *B. henselae* Fizz genotype (identity 527/527 bp) was independently amplified and sequenced from the mitral valve prosthesis removed February 13, 2013, just prior to his death. Based upon blood and tissue PCR amplification and DNA sequencing results, this veterinarian was infected with two *B. henselae* genotypes in January 2012 and the Fizz genotype persisted at the time of his death. When and how these infections were acquired, how long the infections persisted, and if *B. henselae* contributed to his illness, FUO and ultimately his death will never be known.

**Table 2:** *Bartonella* spp. serology and PCR results from blood, serum, BAPGM enrichment blood culture and subculture agar plate swab or tissues from each of the two veterinarians

						Bartonella IFA reciprocal titers					
Veterinarian	Collection Date	Sample	PCR original sample	PCR culture	Subculture isolate	*Bvb* I	*Bvb* II	*Bvb* III	*Bh*HI	*Bh*SA2	*Bk*
#**1**	1/9/2012	FFPE Bone Marrow	*Bh* Fizz	N/A	N/A						
	1/23/2012	Serum	Neg			<16	<16	<16	<16	<16	<16
		Blood	Neg	Neg 7, 14	Neg						
	1/28/2012	Serum	*Bh* SA2								
		Blood	Neg	Neg 7, 14	Neg						
	1/30/2012	Serum	Neg								
		Blood	Neg	Neg 7, 14	Neg						
	4/11/2012	Serum	Neg			<16	<16	<16	<16	<16	<16
		Blood	Neg	Neg 14	Neg						
	4/13/2012	Serum	Neg								
		Blood	Neg	Neg 14	Neg						
	4/16/2012	Serum	Neg								
		Blood	Neg	Neg 14							
	2/13/2013	FFPE Mitral Valve Prosthesis	*Bh* Fizz	N/A	N/A						
#**2**	2/24/2011	FFPE (LN supraclavicular)	Neg	N/A	N/A						
	7/25/2012	FFPE (cervical LN)	*Bh* SA2	N/A	N/A						
	4/5/2013	Serum	Neg	N/A	N/A						
		Blood	Neg	Neg 7, 14	Neg	<16	<16	<16	<16	<16	<16
	4/6/2013	Serum	Neg								
		Blood	Neg	Neg 7, 14	Neg						
	4/6/2013	Serum	Neg								
		Blood	Neg	Neg 7, 14	Neg						
	4/8/2013	Serum	Neg								
		Blood	Neg	Neg 7, 14	Neg						
	unknown	FFPE bone marrow	Neg	N/A	N/A						

N/A- Test not applicable for the sample tested

FFPE- Formalin-fixed paraffin-embedded

*Bh*- *Bartonella henselae*, genotypes SA2 or Fizz

I have been a veterinary internist at North Carolina State University, College of Veterinary Medicine since 1982. As such, I consult with regional veterinarians on a near daily basis. Despite the fact that Veterinarian #2 practiced medicine in North Carolina for 37 years, I did not know him personally; however, as fate would have it, he lived in a house along the North Carolina coast adjacent to a close friend who was familiar with our *Bartonella* research. Thus, it was a mutual friend who suggested *Bartonella* testing. Veterinarian #2 developed lymphadenopathy involving the neck, axillary lymph node (2 cm), and root of the mesentery (radiographically a 4 cm lymph node). On February 24^th^, 2012, the left supraclavicular lymph node was surgically excised at Carolina East Medical Center. Histologically, the lymph node contained a mixed population of small and large lymphocytes, plasma cells, and scattered neutrophils, without evidence of necrosis, granulomatous inflammation, or abscess formation. There were no Reed-Sternberg cells indicative of Hodgkin’s disease. Preliminary pathological diagnosis was lymphadenitis of uncertain etiology. Fungal, bacterial and mycobacterial cultures were negative. Immunophenotypic studies, performed at Johns Hopkins Hospital as part of the standard lymphoma protocol, supported a diagnosis of angioimmunoblastic T cell lymphoma (AILT), with no specific morphologic or immunophenotpic characteristics to allow subclassification. In situ hybridization for Epstein Barr Virus (EBER) identified scattered positive cells. An addendum report from The Johns Hopkins Hospital contained the following statement: “Careful correlation with history is recommended to rule out immunodeficiency. The morphologic features coupled with a high proliferation rate and numerous mitoses are not seen in typical AILT cases; however, additional studies and clinical correlation are needed to exclude this possibility.” March 15^th^, 2012, the patient was referred to Duke University Medical Center where the pathologist concurred with the diagnosis of AILT, however, the attending lymphoma oncology specialist suggested the possibility of self-limiting lymphadenitis. July 17^th^, 2012, a pathologist at MD Anderson Medical Center concurred with the AILT diagnosis. A cervical lymph node, biopsied July 25^th^, had effacement of normal nodal architecture by atypical lymphoid infiltrates, accompanied by mild vascular endothelial proliferation, and scattered eosinophils and plasma cells. Angioimmunoblastic features were less prominent and features of lymphoma were more prominent than the previous biopsy. EBER staining was negative. Immuno-histochemical staining again supported a diagnosis of AILT. The cancer was PCR positive for clonal T-cell receptor gamma gene rearrangement and negative for clonal B-cell immunoglobulin gene rearrangement. 1 Chemotherapy consisted of oral prednisone and five cycles of CHOP (cyclophosphamide, doxorubicin, vincristine, prednisone) therapy. Beginning five months after initiating CHOP chemotherapy, the patient began experiencing frequent rigors, night sweats, and was ultimately diagnosed with FUO, which at various time points was treated with intravenous and orally administered fluoroquinolones, cephalosporins, antivirals, antifungals, intravenous dexamethasone and oral prednisone, all of which failed to induce sustained resolution in the FUO. As described for Veterinarian #1, infection with *Bartonella henselae* can cause FUO [18, 19].

By September 2012, Veterinarian #2 was referred to MD Anderson Center. He was anemic (HB 8.2 mg/dl, thrombocytopenic [89,000 platelets/ul]), and had a lymphocytosis (12,500/ul). A core bone marrow biopsy contained multiple, atypical, lymphohistiocytic aggregates, with a prominent epithelioid component. Immunophenotyping supported AILT with marrow involvement. In October 2012, Veterinarian #2 requested *Bartonella* testing. Because of the historical administration of several antibiotics, BAPGM enrichment blood culture/PCR was likely to be diagnostically less sensitive; therefore, paraffin-embedded lymph node biopsies obtained in February and July 2012 were tested by conventional PCR. *Bartonella henselae* (SA2 genotype) was successfully amplified and sequenced from the July lymph node specimen. Shortly thereafter, treatment with doxycycline as the sole therapy was instituted for bartonellosis and the rigors and fevers temporarily resolved. In completing the study questionnaire, this veterinarian reported being healthy prior to the development of lymphadenopathy in February 2012, and with the exception of FUO accompanied by rigors and night sweats, CHOP (cyclophosphamide, doxorubicin, vincristine, prednisone) chemotherapy had been well tolerated. When the *B. henselae* lymph node PCR result became available, I related our experience with doxycycline therapy in experimentally and naturally infected cats [21], and naturally-infected dogs [22] to the patient’s infectious disease physicians. Doxycycline as a sole antibiotic would suppress *Bartonella* sp. bacteremia, but rarely, if ever, cured the infection. In humans, doxycycline treatments as long as 3 months in duration have resulted in symptomatic and hematological improvement, but sequential blood culture/PCR indicated *B. henselae* infections persisted [13, 23].

Due to the progressive nature of the AILT, a decision was made to treat the patient with allogeneic bone marrow stem cell transplantation; however, this therapy was repeatedly delayed between October 2012 and April 2013 due to recurrent bouts of FUO, which were temporally followed administration of chemotherapy. *B. henselae* infection was not detected by BAPGM enrichment blood culture/PCR [24] in April 2013 (Table 2), shortly before his death on April 21^st^, 2013. The planned allogeneic bone marrow stem cell transplantation was never performed.

In asking the question: “Did *B. henselae* contribute to the deaths of these two veterinarians?”; we would first have to ask, does *B. henselae* cause FUO and endocarditis? The answer is yes. A second important question is: “What microbiological evidence supports *B. henselae* infection?” For Veterinarian #1, *B. henselae* DNA was amplified and sequenced from three diagnostic specimens (bone marrow, blood and mitral valve prosthesis) collected at three different time points between January 2012 and February 2013. For Veterinarian #2, *B. henselae* DNA was amplified and sequenced from a paraffin-embedded lymph node obtained surgically, four months prior to PCR testing in our laboratory. Subsequently, despite ongoing febrile illness, serology and BAPGM enrichment blood culture testing failed to identify *B. henselae* DNA or antibodies. As summarized in a recent review [25], our research laboratory has substantial experience with paraffin embedded tissue PCR [26, 27]. We have also previously described precautions in processing tissues to avoid DNA carryover [28]. Our laboratory protocols describe, and we routinely enforce, rigid work flow patterns to avoid carryover or DNA contamination with amplified products. Also, negative DNA extraction, PCR amplification, and BAPGM enrichment blood culture controls are used routinely to assess *Bartonella* spp. DNA contamination with every sample set processed within the laboratory. DNA carryover and amplicon contamination are considered an unlikely source for the microbiological findings in these two veterinarians. Although isolation is the microbiological “gold standard” for documenting a bacterial infection, PCR amplification of organism-specific DNA sequences is being used to document the presence of fastidious [29], stealth [1] or “unculturable” bacteria from patient samples [30]. PCR amplification of organism-specific DNA sequences does not confirm that the bacteria is viable or that the organism is responsible for the patient’s symptoms or pathology. However, the molecular microbiological evidence supported *B. henselae* infection in both veterinarians.

Another important microbiological question; “Was there serological evidence to support exposure to *B. henselae*?” For reasons that remain unclear, a substantial (50-75 %) subset of *Bartonella* spp. bacteremic patients do not have detectable IFA antibodies [9, 31]. With chronic, asymptomatic or minimally symptomatic *Bartonella* bacteremia, a seronegative status may be the norm, rather than the exception; thus, serology is not a diagnostically or epidemiologically sensitive modality. Often after weeks or months of prior antibiotic therapy, many veterinary workers are tested for *Bartonella* infection as an afterthought or as a “test of last resort.” Using currently available diagnostic techniques, these bacteria are difficult to enrich, to isolate or to PCR amplify from tissues under optimal testing conditions. Long delays before obtaining or processing patient specimens, concurrent or recent antibiotic administration, and the number of blood or tissue samples tested influence the sensitivity of the BAPGM enrichment blood culture/PCR platform. [24, 32] For biopsy specimens, the tissue sample size, the duration of formalin fixation prior to paraffin embedding, and the severity and type of inflammatory response [26, 27] are important factors that determine diagnostic PCR sensitivity. Collectively, these factors influence whether a diagnosis is confirmed or the infection is missed in a given patient. Timelier collection of optimal specimens from these two veterinarians may have resulted in different and diagnostically more beneficial *Bartonella* microbiological findings, than we reported above.

Assuming the possibility that both veterinarians were infected with viable bacteria at the onset of illness and/or when tested, the next question becomes: “Did the bacterium play a role in the initiation or progression of each patient’s illness?” For Veterinarian #1, the approximate two-year history of non-specific, waxing and waning symptoms is consistent with the questionnaire responses reported by other *Bartonella* bacteremic veterinary workers [9–14]. Establishing causation for non-specific symptoms that accompany infectious and non-infectious disease processes is difficult, particularly due to co-morbidities. However, documenting persistent *Bartonella* sp. bacteremia in “non-immunocompromised” individuals is not impossible, as we have repeatedly demonstrated [9–14, 23]. Persistent occult blood stream infection may predispose patients to ongoing microvascular injury, bacterial localization within various tissues including the vascular endothelium and heart valves, and the development of non-specific symptoms. One could hypothesize that Veterinarian #1 progressed from non-specific symptoms to culture-negative endocarditis, to FUO, to infection of the mitral valve prosthesis over a three-year timeframe. Currently, physicians are taught that *B. henselae* infection in immunocompetent people is synonymous with cat scratch disease (CSD), which is considered a self-limiting infection. Although CSD is most often self-limiting, this does not appear to be uniformly true [33]. Performing a sequential, long-term cohort study of CSD patients in the United States, as reported from Israel [33], might identify a subset of persistently *B. henselae* bacteremic patients with associated symptomatology and allow for the documentation of progression of rheumatologic [34] and/or neurologic disease [15].

A biologically more complex question is: “Did *B. henselae* infection predispose to the development of lymphoma?” *Bartonella* spp. can invade numerous host cells, within which the bacteria modify cellular functions by injecting peptides and potentially transporting bacterial DNA into the cell [1, 4, 6, 34]. *B. henselae* contains bacteriophages [35] that might also facilitate DNA translocation events. *Bartonella* spp. appear to play causative or cofactor roles in the development of vasoproliferative tumors in animals [36] and immunocompetent as well as immunocompromised people [6]. Comparative studies of naturally-occurring lymphoma involving animals and humans are warranted to potentially generate evidence that supports comparative infectious disease causation [25]. Although not well studied in human patients, dogs experimentally-infected with *Bartonella vinsonii* subsp. *berkhoffii* became immunosuppressed [37]. Infection-induced immune suppression, as suggested by the pathologist reviewing the original lymph node biopsy from Veterinarian #2, has been suggested to occur in association with human bartonellosis, caused by *B. bacilliformis* [1, 4]. Potentially, DNA translocation events in conjunction with persistent infection-induced immune suppression could predispose a patient to develop lymphoma.

Did *B. henselae* play a role in the deaths of two veterinarians? Perhaps yes and perhaps no. For both veterinarians, it seems likely that *B. henselae* was responsible for FUO. During their illnesses, and after their respective deaths, I communicated with both wives. Based upon their comments during these challenging conversations, it is their hope that some good may come from their husband’s deaths. Both men were outstanding fathers, husbands, veterinarians and community servants, each participating in a large number of volunteer activities. From my perspective, it is time for a “*Bartonella* tipping point,” whereby this emerging pathogen is accorded a higher national (United States) and international research priorities.

## Abbreviations

FUO: Fever of unknown origin
EBER: Epstein Barr Virus
AILT: Angioimmunoblastic T cell lymphoma
CSD: Cat Scratch Disease

## Footnotes

1 NeoGenomics Laboratories, Irvine, Ca.
